# Proteomics as a promising biomarker in food authentication, quality and safety: A review

**DOI:** 10.1002/fsn3.2842

**Published:** 2022-03-24

**Authors:** Muhammad Afzaal, Farhan Saeed, Muzzamal Hussain, Farheen Shahid, Azhari Siddeeg, Ammar Al‐Farga

**Affiliations:** ^1^ 72594 Department of Food Science Government College University Faisalabad Faisalabad Pakistan; ^2^ 119098 Department of Food Engineering and Technology Faculty of Engineering and Technology University of Gezira Wad Medani Sudan; ^3^ 441424 Department of Biochemistry College of Sciences University of Jeddah Jeddah Saudi Arabia

**Keywords:** adulteration, food quality, foodborne pathogens, proteomics

## Abstract

Adulteration and mislabeling have become a very common global malpractice in food industry. Especially foods of animal origin are prepared from plant sources and intentionally mislabeled. This type of mislabeling is an important concern in food safety as the replaced ingredients may cause a food allergy or toxicity to vulnerable consumers. Moreover, foodborne pathogens also pose a major threat to food safety. There is a dire need to develop strong analytical tools to deal with related issues. In this context, proteomics stands out as a promising tool used to report the aforementioned issues. The development in the field of omics has inimitable advantages in enabling the understanding of various biological fields especially in the discipline of food science. In this review, current applications and the role of proteomics in food authenticity, safety, and quality and food traceability are highlighted comprehensively. Additionally, the other components of proteomics have also been comprehensively described. Furthermore, this review will be helpful in the provision of new intuition into the use of proteomics in food analysis. Moreover, the pathogens in food can also be identified based on differences in their protein profiling. Conclusively, proteomics, an indicator of food properties, its origin, the processes applied to food, and its composition are also the limelight of this article.

## Graphical abstract

In this context, proteomics stands out as a promising tool used to report aforementioned issues. The development in the field of omics has inimitable advantages in enabling the understanding of various biological fields especially in the discipline of food science. In this review, current applications and role of proteomics in food authenticity, safety, and quality and food traceability are highlighted comprehensively. Additionally, the other components of proteomics have also been comprehensively described.

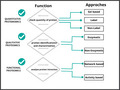

## 1 OVERVIEW

Since 1994, the term “proteomics” was first used and, to date, it has been used in wide applications in analytical chemistry, clinical food microbiology, biotechnology, and food technology. Proteomics is the analysis of proteins at a large scale in a particular biological system at a particular time (Boersema et al., 2015; Pandey & Mann, 2000). It is a very valuable tool in food analysis when applied to different analytical techniques (Marzano et al., 2020). Globally, food trends are drastically changing in production and consumption of food and consumers are paying huge attention to what they are eating. Therefore, knowledge of foodomics is gaining huge popularity in this segment. Foodomics is defined as studies of application of advanced omics approaches to food (Cifuentes, 2017). Foodomics includes epigenetics, transcriptomics, metabolomics, proteomics, peptidomics, and/or genomics to investigate food traceability, food quality, food safety, and also to find new bioactive components in food (Jagadeesh et al., 2017). Proteomics being one of the omics has been extensively used in food research today (Raposo de Magalhães et al., 2020).

In current era, consumer is not only focusing the sensory attributes of the food product. Consumer demand for safe, nutritious, functional, minimal process, and low additives containing food is increasing (Saeed et al., 2021). In this context, proteomics has the promising potential to prove the reliability and safety of the food products. Proteomics are integral in tracking the product from raw materials to finished products. Furthermore the proteomics has great potential for food industry in various areas of quality, traceability, optimization, storage nutrition, and safety. However, it will take some time to adopt such sophisticated tools at industrial scale.

Proteins act as an indicator of origin, properties, and processes conducted on food (Ortea et al., 2016). Furthermore, proteomics can be used to inspect food quality which can be enhanced by improving the processes used in food production (Creydt & Fischer, 2020). However, false claims are being made and mislabeling is done by adding another cheaper alternative ingredient instead of the main ingredient written on label (Moore et al., 2012). Different species of animals are used to make a particular product instead of the one written on the label which is a major point from a religious concern as well. This type of adulteration is a food safety threat as the replaced ingredient may cause allergies or health issues to the consumer (Spink & Moyer, 2011). Specific authentication when done with conventional methods is a time‐consuming task and cannot be applied to detect adulteration of less than 5% of the product (Špoljarić et al., 2013). In this context, proteomics are an effective tool in the detection of adulterants in food (Girolamo et al., 2014). Moreover, proteomic analysis of food is faster and gives in‐depth analysis of food even at peptide level (Gallardo et al., 2013). Proteins are used as markers of different properties, compositions, and origins of food; therefore, knowledge of proteomics is used for this purpose (Erban et al., 2021). The knowledge of proteomics is applied for product traceability, authentication, and protein profiling of food especially for animal‐based products (meat and dairy products) (Leitner et al., 2006; Guarino et al., 2010).

Food safety is an important health concern. Many people across the globe suffer from various foodborne illnesses every year (Bolek, 2020). Sometimes, negligence in food safety concerns can even cause death of the patient in conditions like hemolytic uremic syndrome caused by foodborne pathogens like *E. coli* O157:H7. Proteomic approaches also help in the identification of microorganisms based on variations in their proteome, thus helping in the detection of different types of pathogens in food (Pavlovic et al., 2013; Shiny Matilda et al., 2020). Proteomic assays used to identify the protein must also be present in the database library. Various peptide fingerprint libraries are commercially available. One of these libraries is “spectra bank” containing mass spectral fingerprints of the pathogenic bacteria species and major spoilage causing species from seafood, and includes 120 species of interest in the food sector (Gallardo et al., 2013). Proteomic methods like HPLC and MS/LC‐MS can be used to detect and identify toxins and allergens in food (Martinović et al., 2016; Sangeetha et al., 2020).

Furthermore, high‐quality products can be made by genetic improvements and studying the changes in protein structure, conformation, and posttranslational modifications (PTMs) due to different processes of food production and thus improving the production process accordingly (Pedreschi et al., 2010). Moreover, a large number of proteins are involved in development of tenderness, color, and odor in meat (Jagadeesh et al., 2017; Zapata et al., 2009). These proteins can be identified in food to enhance the quality of food. Multiple techniques‐based knowledge of proteomics has been used for the protein profiling of food, detection of foodborne pathogens, and identification of protein markers, which involve mass spectrometry (MALDI‐TOF and electrospray ionization), HPLC, and gel electrophoresis. This review covers the applications of proteomics in food authentication, quality, and safety using various advanced techniques of food analysis concerned with proteomic approach.

## 2 REVIEW METHODOLOGY

The literature search was carried out using scientific databases comprising Scopus, Science Direct, Google scholar, PubMed, Cochrane Library, Science Hub, and Library genesis using the following subject headings proteomics, food authentication, food safety, and biomarker using keywords: "Proteomics as analytical tool, proteomics in food authentication and food quality, adulteration, Foodborne pathogens and their identification, foodborne pathogens and toxins." The authors collected the latest available literature from primary and secondary sources.

## 3 PROTEOMICS

Proteomics is proteins study at a very large scale. Marc Wilkins in 1994 first use the word proteomics. A proteome is known as a complete set of proteins expressed or produced by a system or organism. Proteomics consists of six classes (Carbonaro, 2004): functional proteomics, expression proteomics, protein–protein interactions, proteome mining, posttranslational modifications, and structural proteomics. It includes the quantitative analysis of a proteome and its protein profiling. Mainly in food sector, proteomics‐based techniques are used for authentication of food products for food safety by identifying foodborne pathogens based on variations in their proteome, allergens, and toxins detections, for process validation and optimization, identification of bioactive compounds in functional foods, and for identification of specie‐specific biomarkers to authenticate meat and dairy products. Proteomics is an effective approach to identify protein as well as the interactions of protein with other components of foods (Kvasnicka, 2003). Mass spectrometry‐based approaches like MALDI‐TOF combined with gel electrophoresis and other nongel‐based techniques are used in proteomic analysis of food products. Proteomics has vast application potential in different industries including food, feed, health, and medicine. Medical research has better option for diagnostic of various health maladies. Proteomics has bright future for its wide spared use in food (safety and nutrition) and other allied fields. However, there are various constraints in use of proteomics like lack of validation, standardization, and most importantly complexity in analysis. Proteomics also helps to enhance the quality of food products by studying the effect of different processes on food proteins, thus improving the food processing line. Classification of proteomics is given in Table 1.

**TABLE 1:** Classes of proteomics

No.	Classes of proteomic	Reference
1	Expression proteomics	Carbonaro (2004)
2	Protein–protein interactions	
3	Functional proteomics	
4	Structural proteomics	
5	Proteomic mining	
6	Posttranslational modification	

## 4 WORKING PRINCIPLE

The working principle of proteomics consists of the following important steps: (i) protein extraction; (ii) protein or peptide separation and quantification; (iii) protein identification; and (iv) data analysis and interpretation.

**FIGURE 1:**
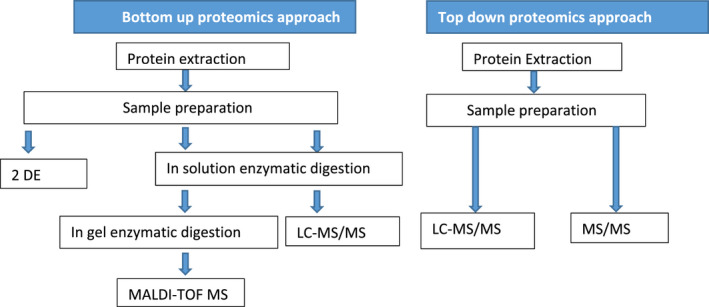
Proteomics workflows for bottom‐up and top‐down proteomics approaches

The protein extraction is done from the sample used for analysis (Gallardo et al., 2013). In the case of complex samples, partial purification, selective enrichment, or depletion of high abundance proteins is also done (Pedreschi et al., 2010; Surinova et al., 2011). The separation of proteins is done using two‐dimensional gel electrophoresis method. Both these separation methods are done in a bottom‐up proteomic approach (Panday & Mann, 2000). The second approach is the top‐down approach in which the digestion step is not done and the peptides from fragmented proteins are directly subjected to mass spectrometry (McLafferty et al., 2007 *)*. After protein separation and digestion of proteins, the quantification and identification of proteins are done by using mass spectrometric techniques (Gallardo et al., 2013) like LC‐MS, MALDI‐TOF MS, or MS/MS. MALDI‐TOF MS is, however, used in protein identification while MS/MS or LC‐MS/MS can be used for identification, quantification, as well as protein characterization. The approach in which 2D electrophoresis is used and afterward followed by mass spectrometric analysis in‐gel digestion of protein is done, which is also called peptide mass fingerprinting. For both these approaches, the protein to be identified is matched with the protein in the database after being subjected to the mass spectrometer. Provided that the corresponding protein is not present in the database, the most homologically related protein is matched (Gallardo et al., 2013). Proteomics workflows for bottom‐up and top‐down proteomics approaches are shown in Figure 1.

## 5 EXPERIMENTAL AREAS

**FIGURE 2:**
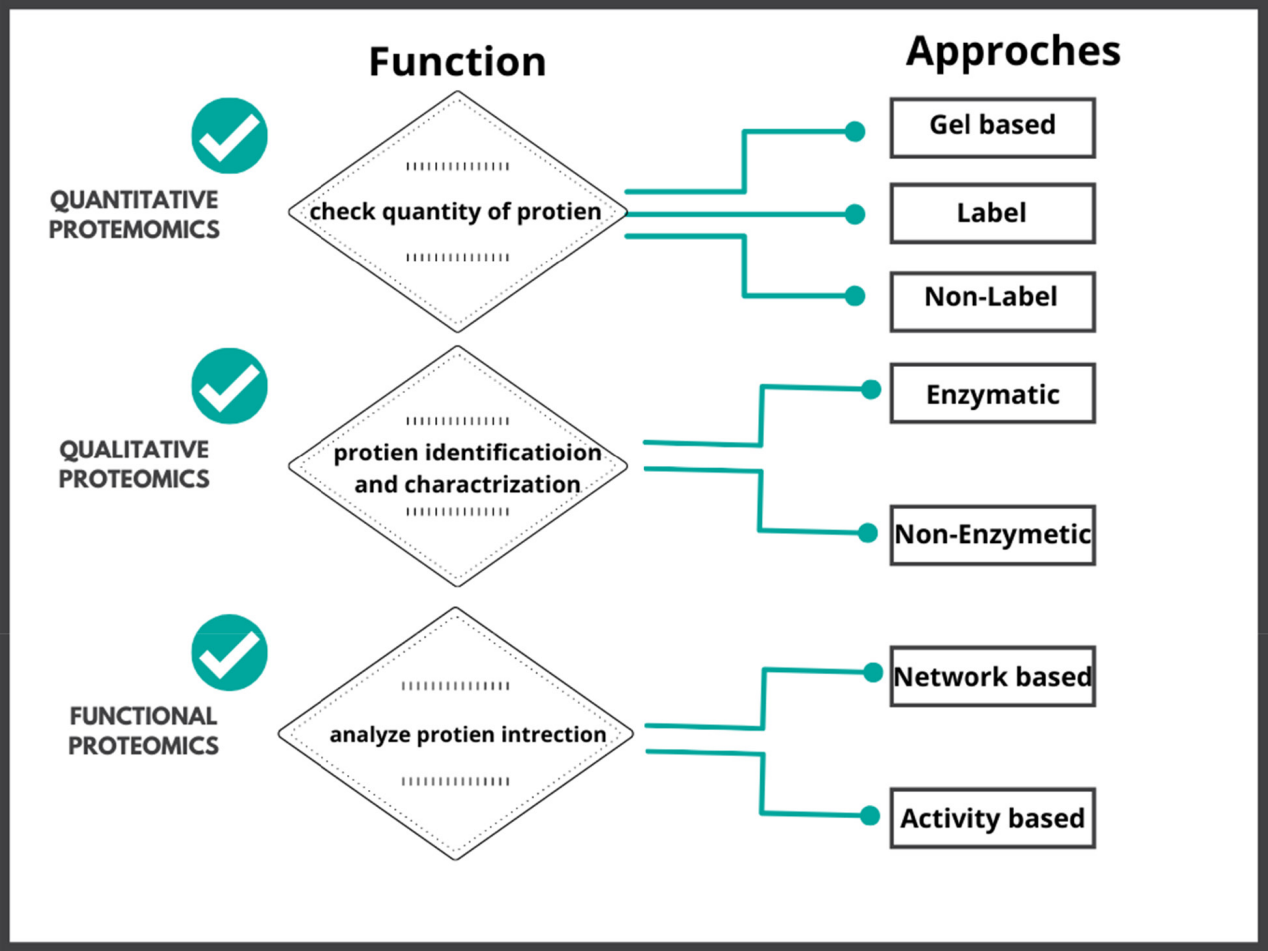
Proteomics experimental areas, their functions, and approaches

Experimental areas that can adopt three key methods in proteomics based on the scientific question to be answered include qualitative, quantitative, and functional proteomics. Proteomics experimental areas, their functions and approaches are also discussed in Figure 2.

### 5.1 Quantitative proteomics

The relative amount of protein in food proteomes can change mainly because of the composition of the food, the biological variability of the food components, and the technical processing of the food. Protein concentrations are determined accurately, and quantitative proteomics requires relative quantification of specific proteins between various samples and absolute quantification. While searching for differences between different conditions based on different treatments, GM or non‐GM food products, quantitative information at the protein level (absolute protein amount or the relative abundance of a particular protein between different samples) can be very helpful. Natural variations in raw materials, technical processing, and storage are common application studies on changes in the food proteome (Restani et al., 1997). Gel‐based methods consist of the comparison of protein abundance determined between different samples as the spot volume and the two‐dimensional electrophoresis (2‐DE) separation of proteins. Using 2‐D fluorescence difference gel electrophoresis (DIGE), protein quantification, authentication, and detection of different adulterants were assessed (Minden et al., 2009). Preseparation gel base is not required in many cases and relative quantification for primary amines is achieved by using labeled mass tags (Boersema et al., 2009), such as dimethyl labeling, isobaric absolute and relative quantification tags (ITRAQ) (Ross et al., 2004), and tandem mass tags (TMT) (Thompson et al., 2003). Label‐free quantification uses multiple assessment methods (Neilson et al., 2011) that take either the spectral counting based on counting the number of peptides assigned to a protein or the area under the curve based on precursor ion spectra peak area in an MS or MS experiment. Quantitative proteomic methodologies have been greatly improved (Gallien et al., 2011) by the implementation of selected reaction monitoring experiments (SRM), a highly sensitive LC‐MS or MS acquisition mode is widely used in biomedical research to validate and verify candidate biomarker proteins.

### 5.2 Qualitative proteomics

The type of proteomics includes the characterization and detection of protein in food products and can include either all the proteins or specific subsets of proteins of particular interest, which is known as qualitative proteomics. Examples of qualitative proteins include glycolytic enzymes in meat or food allergens and caseins in dairy products. The two most common protein identification methods include Peptide Fragment Fingerprinting (PFF) and peptide mass fingerprinting (PMF) that are both involved in enzymatic digestion of the identified proteins. In a process named top‐down proteomics (Lafferty et al., 2021), tandem mass spectrometry (MS/MS) data from the intact protein can be used alternatively. All of these procedures include the sequence information present in the database of the homologous or respective protein. Protein databases currently available, such as UniProtKB (Uniprot Consortium, 2019) or NCBI (National Center for Biotechnology Information), U.S. National Library of Medicine, Bethesda, MD, USA, mostly do not provide accurate protein sequence data for the multitude of proteins found in food products of various plant, animal, or fungal origins. PTMs may occur owing to food processing methods and a wide variety of biological signals, including preservation treatments and cooking. Only a few forms of PTMs are being studied extensively out of 300 different forms (Zhao & Jensen, 2009) such as glycosylation, acetylation, oxidation, and phosphorylation. Nonbiological PTMs, such as aromatic hydroxylation, Maillard glycation, thiol oxidation, carbonylation, condensation, peptide backbone breakdown, and side chain removal, frequently occur during food storage and processing, which are called nonenzymatic PTMs (ne‐PTMs) (Clerens et al., 2012; Pischetsrieder & Baeuerlein, 2009).

### 5.3 Functional proteomics

Protein‐to‐protein interactions and protein interactions with other molecules including the effects of concerned interactions are studied in functional proteomics (Coombs, 2020; Kiemer & Cesareni, 2007). Protein profiling activity‐based probes of inhibitor screening and active enzyme levels are used in functional approaches (Serim et al., 2012). Another similar functional proteomics is activity‐based proteomics, which studies the basic activities of proteins in a sample, such as inhibition and function (Elmore et al., 2021). Mass spectrometry imaging, a new imaging mode that enables proteins to be mapped within a tissue or sample section, has proven to be a tool for functional proteomics, as it can help to understand their functions by locating the different protein isoforms (Angel & Caprioli, 2013).

## 6 PROTEOMICS APPROACHES IN FOOD AUTHENTICATION

Consumers’ demand regarding food authentication and true food labeling is becoming trendier because of health, nutrition, and religious concerns (Meijer et al., 2021). However, with increasing food demand, adulteration and false labeling have become a huge concern in the food chain and have become difficult to monitor food quality. Proteomics methods have currently been utilized as a quicker, adaptive, and high‐throughput outlook for assessing the validity and traceability of species in food products due to recent developments in MS (Piñeiro et al., 2003). Therefore, proteomics has been used as a part of multiple studies for the adulteration detection, quantification, and identification in food products by identification and detection of specie‐specific protein markers and the protein markers of different processes on food with the help of mass spectroscopic techniques (LC‐MS, tandem mass spectrometry, and MALDI‐TOF MS). These proteomics‐based techniques have been applied to milk and dairy products, meat, and plant‐based foods for product traceability and authenticity (Ortea et al., 2016). MS is used in reference samples for both the identification of species‐specific peptide fingerprints and the detection of certain diagnostic peptides in actual samples (Carrera et al., 2007). Proteomics tools take advantage of MS high‐throughput ability to achieve rapid, reliable, and responsive detection, characterization, and quantification of peptides and proteins. The complexity of the sample treatment was not ideally suited to high‐throughput analysis; proteomics‐based methodologies are automated and implemented partially described species in genomic databases.

### 6.1 Proteomic approaches to study protein–protein cross‐linking in food

Protein–protein cross‐linking can be described as covalent bonding between intramolecular protein polypeptides or amino acid residue within intramolecular protein polypeptides (Feeney & Whitaker, 1987). Generally, cross‐linking is divided into three categories that may occur in food protein: (1) natural ones that are present in raw material before processing; (2) those that are intentionally added by cross‐linking reagents which could be enzymatic or chemical; and (3) those that are created by processing or environmental disruptions, such as UV exposure, heat treatment, pH, and drying changes. Cross‐linking affects the nutritional (Friedman, 1999a, 1999b) and functional (Singh, 1991) properties of food. Proteomics involves the characterization and detection of proteins arrangement of research areas, along with the study of the location and structure of proteins expressed under specific conditions at a given time. Typical applications involve the identification of the primary structure of the protein (Ishihama et al., 2005), protein and peptide analysis, protein quantitation, and the characterization of modifications in posttranslation. Proteomic methods include bottom‐up or bottom‐down cross‐links that cause issues in both. It is possible to explain classical bottom‐up proteomics broadly in four major steps. Firstly, techniques including two‐dimensional polyacrylamide and electrophoresis (2D‐PAGE) are used to extract and process the protein from biological matter. Secondly, proteins usually with trypsin are digested into peptides with proteases (Olsen et al., 2004). Thirdly, a combination of liquid chromatography (LC) and 2D‐PAGE, the complex peptide mixture, may be isolated before being analyzed by a mass spectrometer. Lastly, the characterization of the proteins and their posttranslational modifications are followed by MS/MS database search (Eng et al., 1994; Pappin et al., 1993; Perkins et al., 1999; Wu & MacCoss, 2002).

### 6.2 Milk and dairy products

Milk is universally important for humans for a lifetime because of the nutrients it provides to the body (Roncada et al., 2012). Therefore, milk quality is an important factor in the determination of milk properties and safety aspects. Milk proteins play a vital key role in providing functional and structural properties to milk. For example, caseins act as an integral part of milk composition contributing to the formation of micelles forming milk fat globule membrane (MFGM) (Roncada et al., 2012). Therefore, since the last two decades, proteomics has become an essential research parameter for scientists to categorize biomarkers for milk analysis (Roncada et al., 2012). Thus, proteomics is a valuable tool in food authentication and traceability as spectroscopic techniques like ESI MS and MALDI‐TOF have been evident to be efficient and time‐saving procedures in the identification of milk protein markers that are specie specific and also in identifying the protein markers that show the application of different processes on milk. Sometimes, the adulteration of goat cheese can be done by mixing cow milk in it which affects the quality of cheese (Agregán et al., 2021). The reference assay for identification of the presence of cow milk in goat cheese or ewe on IEF gel is based on the identification of the bands γ2‐ and γ3‐caseins of cow milk (Ortea et al., 2016), and the identification of cow caseins has been proved by this assay (H.K. Mayer, 2005; J. Špoljarić et al., 2013). But the detection of proteins is only possible in samples with at least 5℅ cow milk. So, MS‐based methods like ESI MS and MALDI‐TOF MS are applicable for such samples and analysis is done faster by these methods. MALDI‐TOF MS can be used for detection of adulteration of bovine milk to buffalo milk or eve. The detection of adulteration up to 0.5% of goat milk with cow or buffalo milk is possible with MALDI‐TOF MS. MALDI‐TOF can be used in the identification of adulteration of raw milk with UHT milk as well. Abd El‐Salam (2014) has reviewed the use of HPLC‐coupled MS and MALDI‐TOF for the detection and quantification of milk contaminants and adulterants. Sometimes, skimmed milk powder is adulterated with soy and pea proteins. This type of adulteration can also be detected by MS/MS technique.

### 6.3 Meat

Global meat production and consumption have increased and coupled with an ever‐growing population, there is concern among governmental bodies and industries that such a high demand may be impossible to meet. Therefore, adulteration of meat with plant‐based protein or less expensive meat of another species is often done nowadays. Proteomic techniques are a fast, reliable, and efficient way of detecting and quantifying such adulterations and finding out the origin of meat. The differences among protein patterns of cattle, chicken, duck and goose were investigated by Montowska and Pospiech (2012) and Montowska and Pospiech (2013, respectively. Soybean proteins added to the processed meat‐based identification are done by using a 2D LC‐MS/MS‐based method (Leitner et al., 2006). The protein glycinin G4 subunit A4 was detected in all meat samples adulterated with soybean protein. This protein therefore can act as a protein marker of adulteration of meat with soybean proteins. Sentandreu et al. (2010) reported a proteomics‐based technique using LC‐MS/MS for identifying chicken in meat mixes. The proteome of meat of two different breeds of pigs was studied and proteins were identified by mass spectrometry in which 1125 proteins were identified and the different proteins were 63 in both breeds. Proteomics has been proved to be a valuable tool in identifying species‐specific peptides/proteins of meat, thus helping in the traceability of meat and detection of adulteration of meat with proteins from other sources and species (Hollung et al., 2009; Murgiano et al., 2010). Proteomics methods have also been helpful for the validation of halal meat from a religious point of view (El Sheikha et al., 2017; Hossain et al., 2020) as adulteration is sometimes done by mixing the meat of different species or selling the meat or meat products of a haram animal in the name of a halal species (Jannat et al., 2018). It can also figure out whether the method used for the slaughtering of animals is halal or not. Techniques like ELIZA and PCR have been used for the identification of protein and DNA molecules that are species specific, respectively. SDS‐PAGE, IEF, ELIZA, and HPLC have been used for the protein‐formed verification of halal food (Amid & Samah, 2019).

### 6.4 Safety aspects of genetically modified organisms (GMOs)

The spontaneous effects of transgenesis in GMOs have raised food safety concerns. Therefore, the knowledge of proteomics has also been applied to the authentication of GMOs. Labeling requirements for GMOs have been implemented in more than 40 countries for food safety concerns and consumer knowledge (Gruère et al., 2007), therefore, it is necessary to have the inspection techniques for GM foods to provide correct information. “Substantial equivalence” is used in the inspection of the safety of GM foods (Pedreschi et al., 2010). The properties and attributes of GM food and traditional food are compared for analysis and assessment (Cellini et al., 2004; Kuiper et al., 2001). GM food safety assessment is done mainly to prove that GM food is safe to consume and will not cause any harm to the consumer (Pedreschi et al., 2010). Proteomics techniques incorporated with LC‐MS and 2DE have been utilized to detect whether the food is GM or non‐GM food. Luo et al. (2009) used a gel‐free proteomic approach combined with isobaric tags for relative and absolute quantitation (iTRAQ) labeling that showed the difference in expression of different proteins between wild‐type rice and GM. A label‐free LC‐MS workflow was shown by Mora et al. (2013) for the relative quantification of proteins in GM and non‐GM tomato varieties. 2‐DE and iTRAQ were used to quantify the differences in proteomes of GM maize and nontransgenic maize in maize seeds (Tan et al., 2017). As a result, of 148 differentially expressed proteins, 106 were in higher numbers in non‐GM maize and 42 proteins were higher in GM maize. Moreover, Liu et al. (2018) used the iTRAQ approach to analyze proteomic profiles in GM‐modified and natural genotypic soybeans (non‐GM). They concluded in their study that the difference in protein expressions in non‐GM soybean species was higher than and different from those caused by GM. Thus, omics‐ and proteomics‐based techniques can play a functional role in identifying genetically modified food in a very cost‐effective manner (Jain et al., 2019).

### 6.5 Seafood

Seafood is traded all over the world and there are bigger chances of adulteration and mislabeling of seafood due to the closely related species of fish and other kinds of seafood. Therefore, seafood authentication and origin are a huge concern to ensure product transparency. For example, an expensive species may be replaced with a cheaper species and sold by the name of the expensive one (Ortea et al., 2016). For this reason, international laws and regulations have been implemented regarding the labeling of seafood. According to the European legislation (European Parliament and European Council, Regulation (EU) No 1379/2013), the label of the seafood must include the commercialized name of the species, the type of methods used to produce it, and the place where the fish or the seafood species was caught or farmed (Ortea et al., 2016). So, there must be reliable methods to analyze the seafood and its products and proteomic methods have been applied for the authentication of seafood. The protein fingerprint database of 54 commercial fish was made by Stahl and Schröder (2017) using MALDI‐TOF technique and the assay was proved successful as 188 unknown samples were identified by it. Species‐specific identification of the fish and fish products is also possible with this technique. Ortea et al. (2010) used native IEF combined with MS or LC‐MS methods for the identification of species‐specific proteins in shrimps and prawns to differentiate between shrimps and prawns. The identification was successful.

Proteomic techniques in the variations in the proteome of organisms of the same species have also been studied. López et al. (2002) studied the quantitative variations in the proteomes of two species of mussels using 2‐DE and PMF analysis. But they concluded that there can be many reasons for these variations; for example, environmental factors or genetic mutations. An assay based on MALDI‐TOF was introduced for the identification of species‐specific protein markers in 25 different species of fish which proved successful and also worked for fish products (Mazzeo et al., 2008). They also found that parvalbumins were the main protein markers and allergens in fish. This proved to be the fastest technique for species‐specific analysis of protein markers in fish. Some proteomic approaches have also been combined with other assays for species‐specific authentication of fish and fish products that are heat processed. Differentiation between species of fish including gadoid fish (Piñeiro et al., 1998), hake species (Piñeiro et al., 2001), and flatfish (Piņeiro et al., 1999) has also been done with the help of qualitative profiling of water‐soluble proteins using 2‐DE. The protein marker found was parvalbumin and as parvalbumin is heat resistant, this technique may also be applied for heat‐processed products.

In a study using label‐free and dimethyl labeling quantification, LC‐MS/MS‐based techniques were used to find the variations between the protein profiles of wild and farmed gilthead sea bream. As a result of farmed fish, the variations were found in the quality of sarcoplasmic proteins, and parvalbumin was more expressed (Piovesana et al., 2016). Mazzeo and Siciliano (2016), for the authentication of fish species in their study on proteomics, reported several methods which can be used for the fishery products identification using proteomics. In their study, they concluded that MALDI‐TOF molecular profiling strategies can lead to fish species identification within minutes, whereas MS proteomics techniques can not only help to identify fish species but also in the identification of major fish allergen (β‐PRVBs). Hence, concluding that MS‐based methods hold the potential to get authentic results in a short time.

### 6.6 Proteomics in food quality

Identification and authentication of food products from farm to fork is getting huge attention from industrialists and consumers. Knowing food composition can not only help to provide clear information to consumers but also help to improve food quality. Proteins, therefore, act as markers of food composition, origin, and processes done on food (Ortea et al., 2016). Thus, the knowledge of proteomics can help in enhancing the quality of food by optimizing the food production process, studying the effect of different processes on proteins in food, and identifying such proteins modified by processing conditions (Pedreschi et al., 2010; Renzone et al., 2021).

### 6.7 Proteomics in process optimization and validation

The different processes in food production affect the quality of food and thus bring changes to the proteins. These changes help food processors to improve the production process by studying the effects of changes brought by a particular process on proteins. Each process during food production brings specific changes to particular marker proteins which act as an indicator whether the process is done properly or not. For example, product quality can be affected negatively by improper heat processing. Protein denaturation and Millard reactions are the major changes caused by heat processing. Allergies against milk products can be induced by carbonylation of b‐lactoglobulin and other milk proteins during industrial processing (Gašo‐Sokač et al., 2010). Therefore, MALDI‐TOF MS is used to detect these carbonylated proteins. Proteins also determine the physicochemical properties and nutritional quality of food; hence, some proteins are involved in color, odor, and tenderness of meat. Some proteins (enzymes) involved in oxidative metabolism are involved in color development of meat. Some proteins like myosin, actin, tubulin, and desmin are involved in beef tenderness (Zapata et al., 2009). These proteins can be detected in meat to ensure the quality of meat in terms of tenderness. Meat quality depends on many different factors including the post mortem factors or modifications in meat proteins. One of the chemical degradations of proteins is dimidiation in which glutamic acid or aspartic acid are produced by hydrolysis of glutamine or asparagine, respectively; the mass spectrometric methods can be applied in the detection of such sort of protein degradation (Ortea et al., 2016; Schmid et al., 2001). Therefore, the outcome of different processing methods on food proteins can be found by the proteomic analysis of food, thus helping in modifying the production process accordingly and showing the validity of a particular process.

### 6.8 Proteomics in postharvest technology

Proteomics approaches can help to improve the postharvest techniques as well. The postharvest losses of vegetables and fruits in developed countries are 10%–30% and are above about 30%–50% in developing countries per year (Legard et al., 2000; Mathabe et al., 2020). The identification of protein indicators of harvest maturity was reported by Abdi et al. (2002) and also the identification of protein indicators of horticultural quality was done (Lee et al., 2006). After harvesting, the harvest is exposed to different stressful conditions that involve cold storage and modified atmosphere storage which leads to different physiological disorders and changes in it (Chrysargyris et al., 2018). These stressful conditions also cause changes to the proteins in fruits and vegetables which act as indicators to detect the particular processes that cause such changes. Thus, they help in improving the postharvest technology. The changes in proteins of citrus fruits upon postharvest storage were reported by Lliso et al. (2007). Low‐temperature storage leads to the formation of induced proteins that are antifreeze. Currently, studies have been done on the expression of genes and accumulation of proteins in noninjured tissues of fruit during postharvest storage (Feng et al., 2016; Marondedze, 2017). Chilling injury in tomatoes revealed the presence of two thioredoxin peroxidase, cold stress proteins, and an RNA‐binding protein in the noninjured part of the tomatoes (Vega‐García et al., 2010). Therefore, if such proteins are manipulated, then these technologies and knowledge can benefit the frozen fruit and vegetable industry (Galindo et al., 2007).

### 6.9 Role in cereals and cereal‐based products

Proteomics has proved to be quite useful to improve the quality of cereals and cereal products (Alves et al., 2019). Proteins that are involved in rice quality and flavor can be identified by studying the proteomes of low‐ and high‐quality rice cultivars (Kim et al., 2009); Bahrman et al. (2004) and Grove et al. (2009) have studied the effects of several levels of sulfur and nitrogen on gluten proteins by using proteomic approaches. Several cold‐responsive proteins were identified by Yan et al. (2006) by the proteomic identification of rice leaves that were given a chilling treatment. The concentration and composition of proteins determine the quality of durum wheat pasta (De Angelis et al., 2008). In addition to this, stress conditions and temperature also affect the protein content and composition in cereals (Juhász et al., 2020). The high temperature tends to alter the composition of protein during grain filling, therefore the flour and the products resulting from such grains will have changes in their properties. Proteins that change by heat stress (Majoul et al., 2003, 2004) have been identified using proteomic methods. Yahata et al. (2005) identified heart‐specific proteins that were used as markers to find cultivars that were best in the making of flour (Yahata et al., 2005). Protein composition helps to determine flour quality (Dupont & Altenbach, 2003; Skylas et al., 2000). Therefore, heat stress during the grain filling can affect the composition of gluten proteins (Hurkman et al., 2013) which increases the size of gluten polymer. Different proteomics techniques used in sea foods, postharvest, and cereal are discussed in Table 2.

**TABLE 2:** Different techniques used in proteomics and their achieved targets

Food groups	Techniques used in proteomics	Objectives of main analysis	Main target of techniques
Shellfish	Native IEF 2‐DE 2‐DE and PMF	Differentiation of shrimp species Discrimination of two scallop populations Differentiation of shrimp species	Sarcoplasmic calcium‐binding protein Mantle proteins Arginine kinase
Gelatin	MS/MS DDA +PFF	Species used	Collagen
Fish	2‐DE 2‐DE, PMF, and MS/MS	Differentiation of tuna species Discrimination of two river fish species	Muscular proteins Triose phosphate isomerase
GMOs	2‐DE 2‐DE DIGE	Comparison of GM and non‐GM maize Comparison of GM and non‐GM soybean Comparison of GM and non‐GM common bean	Kernel proteome Leaf proteome Grain proteome Leaf proteome Seed proteome Grain proteome

### 6.10 Proteomics in food safety

The knowledge of proteomics has also been applied to food safety. By using proteomic techniques, food spoilage microorganisms (Gallardo et al., 2013) and different foodborne pathogens can be identified based on changes in their proteome (Carrera et al., 2020; Pavlovic et al., 2013). Food allergens have also been studied to be identified by proteomic techniques. MALDI‐TOF MS, MALDI‐TOF, and HPLC ESI MS/MS are some efficient proteomic‐based techniques that have been used in several studies for detection, quantification, and identification of different microbes, their toxins, and different allergens in food.

### 6.11 Identification of foodborne pathogens and toxins

Proteomic techniques can be used for the quantification, identification, and detection of foodborne pathogens. More than 250 recognized pathogens are known to cause foodborne illnesses, mainly microbes and their toxins. Although morphological, biochemical, and DNA methods are used to identify and classify microorganisms, proteomic methodologies are being applied to help identify the food spoiling foodborne pathogens and microorganisms. For this purpose, new technologies to detect and classify microorganisms accurately and rapidly, such as the new MS‐based proteomics tools, complement classical and genetic‐based identification techniques. Proteomics technologies, primarily in clinical microbiology, biodefense, and environmental science, have been used in regular bacterial identification. In the field of microbial food MS, however, little work has been carried out to classify foodborne microorganisms and pathogens responsible for food spoilage. For the detection of 24 different food spoilage bacteria and foodborne pathogens, including genera such as Staphylococcus, Yersinia, Proteus, Escherichia, Lactococcus, Listeria, Pseudomonas, Morganella, and Salmonella, MALDI‐TOF MS of intact bacterial cells was used (Mazzeo et al., 2006). In several studies, matrix‐assisted laser desorption ionization–time of flight (MALDI‐TOF MS) has been used for the identification of foodborne pathogens especially bacteria. Different foodborne pathogens like *listeria* and *Escherichia coli monocytogens* (Jadhav et al., 2014) can be detected and identified by this time‐saving and cost‐effective technique (Singhal et al., 2015). Based on the profiling of the whole bacterial proteome, MALDI‐TOF MS is providing a fingerprint specific to the analyzed microorganisms in that specific time and physiological condition (Pavlovic et al., 2013). The fingerprint obtained through this method has many applications such as the characterization of subspecies, strains, and serovar and is specific to the analyzed microorganisms (Piras et al., 2016). Shiga toxin‐producing *Escherichia coli* (STEC) is linked increasingly to major outbreaks of food‐borne illness, identified by Fagerquist et al. (2014) by using tandem mass spectrometry and MALDI‐TOF. Up to one colony‐forming unit (CFU) of *L. monocytogens* per ml can be identified by MALDI‐TOF MS within 30 hr. *Staphylococcus aureus* causes illness in almost 185,000 people in the USA annually. A mass spectrometry‐based assay was used by Callahan et al. (2006) to characterize one of its toxins (staphylococcal enterotoxin B). Toxins cannot be destroyed by common food processing techniques. Proteomic techniques like LS‐MS/MS have also been used in the detection of toxins like mycotoxins and aflatoxins in food. Martinović et al. (2016) reviewed the use of proteomics for identification of toxins.

### 6.12 Allergens detection

Allergens are the agents which elucidate the body responses. Allergen increases the histamines, immunoglobulin‐IgE, cytokines, and other body responses that cause allergy (Jagadeesh et al., 2017). Food allergens are also a big problem in food safety as there is no cure for allergy and the only way to prevent it is to avoid those foods one is allergic to. Allergens in food can be detected by proteomics‐based techniques. Carrera and colleagues (Carrera et al., 2012) used MS/MS (LIT) mass spectrometer to identify parvalbumin fish allergen in less than 2 h. The identification of allergens is also possible with mass spectrometric methods either gel‐based or nongel‐based HPLC combined with tandem mass spectrometry. There are six main food allergens. Ninety percent of the food hypersensitivity is due to three plant‐based food allergens found in peanut, soy, and wheat (Natarajan et al., 2006; Šotkovský et al., 2008). In allergenomics, immunoblotting of IgE‐reactive proteins is done using a serum of allergic patients using 2‐DE (Akagawa et al., 2007). Proteolytic processing of peanut allergens (Ara H 3 and its isoallergens) has been studied using proteomics‐based techniques (Piersma et al., 2005). Allergens from processed peanuts have been identified using a proteomic‐based assay (Chassaigne et al., 2007). The postharvest technique known as controlled atmosphere storage has been shown to change the quantities of allergenic proteins in fruits causing birch pollen allergy (Pedreschi et al., 2007; Sancho et al., 2006). The amounts of 10 different allergens in soybean were found by Houston and colleagues using a label‐free proteomic method. Proteomic approaches to assess authenticity of different food products are presented in Table 3.

**TABLE 3:** Proteomic approaches to assess authenticity of different food products

Food	Technique	Purpose of analysis	Target	References
Milk and milk products	IEF MALDI‐TOF MS protein/peptide	Milk adulteration Milk adulteration Adulteration of milk powder with pea and soy proteins Cheese adulteration	Caseins Low Mr proteins (<25KDA)	Di Girolamo et al. (2014); Sassi et al. (2015)
Meat	2‐DE 2‐DE DIGE MALDI‐TOF MS Peptide profiling MS/MS DDA	Differentiation of meat species (cattle, pork, chicken, turkey, duck, and goose) Differentiation of two Norwegian breeds Species identification (32 mammal species) Detection of chicken meat in meat mixes	Myosin light chains Muscle water‐soluble proteins Collagen Myosin light‐chain 3	Montowska and Pospiech (2012), Montowska and Pospiech (2013).
Wine	MALDI‐TOF MS fingerprinting	Discrimination of white wine varieties Classification of Croatian white wines	Wine protein and peptides Caseins	Rešetar et al. (2016), Cereda et al. (2010)
Honey	MALDI‐TOF MS protein profiling 2‐DE	Geographical origin Floral origin	Water‐soluble honey proteins	Wang et al. (2009), Di Girolamo et al. (2012)

As the proteomic methods applied for the identification of allergens can be gel‐based methods or gel‐free methods that are usually combined with mass spectrometric techniques like HPLC‐MS/MS (in case of gel‐free techniques) or 2D immunoblotting and MS (in cases of gel‐based methods). Several gel‐based proteomics studies have been done for the allergen's identification in different foods. These studies used 2‐DE for detecting and identifying allergens in different foods. Picariello et al. (2015) studied wheat beer for the detection of allergens in it. Apostolovic et al. (2014) studied the immunoproteomics of processed beef to find allergens in it. In 2015, Odedra et al. studied the allergy through milk in adults and children. Moreover, Hettinga et al. (2015) studied the proteomes of allergic and nonallergic mothers’ breast milk. Goliáš et al. (2013) studied the detection of allergens in rice and Tomm et al. (2013) studied the allergens in fish.

## 7 CONCLUSION

Proteomics is the potential analytical approaches to classify the safety and quality changes during the storage of food commodities. Proteomics has various applications in determining the authenticity, adulterants, and toxicity in food products. Various challenges exist in predicting the safety and quality of the products in terms of accuracy. Conclusively, the application of proteomics is an emerging technology that can be helpful to ensure high‐quality safe foodstuff.

## CONFLICT OF INTEREST

All authors declare that they have no conflict of interest.
